# Genome-wide identification of the SBP-box gene family in banana and functional characterization of MaSBP17 as a negative regulator of fruit ripening

**DOI:** 10.3389/fpls.2026.1879074

**Published:** 2026-07-02

**Authors:** Shiyu Lu, Yu Yang, Weijun Xiao, Zhiwei Jia, Yulin Hu, Yajie Duan, Zhencai Pang, Dequan Sun, Zhiwei Lu, Huigang Hu

**Affiliations:** 1 State Key Laboratory of Tropical Crop Breeding, South Subtropical Crops Research Institute, Chinese Academy of Tropical Agricultural Sciences, Zhanjiang, China; 2 Chinese Academy of Agricultural Sciences, Beijing, China; 3 National Key Laboratory for Germplasm Innovation and Utilization of Horticultural Crops, Huazhong Agricultural University, Wuhan, China; 4 Key Laboratory of Tropical Fruit Biology, Ministry of Agriculture & Rural Affairs, Zhanjiang, Guangdong, China

**Keywords:** banana, fruits ripening, MaSBP17, SBP, transcription factors

## Abstract

**Introduction:**

Banana (*Musa* spp.) is a primary climacteric fruit characterized by a rapid post-harvest ripening process that limits its shelf-life. Although SQUAMOSA promoter-binding protein (SBP) transcription factors are vital regulators of plant development, their roles in banana ripening remain largely undefined.

**Methods:**

In this study, we identified 57 *MaSBP* genes across the *Musa acuminata* (v4) genome, categorized into six distinct subfamilies (Groups I-VI). We further performed expression profiling, RT-qPCR analysis, and functional validation via virus-induced gene silencing (VIGS) to characterize these genes.

**Results:**

Our analysis reveals a significantly expanded family with diverse gene architectures and physicochemical properties. We identified several ethylene-responsive *MaSBPs*, with *MaSBP17* exhibiting a significant downregulation during the climacteric phase. *MaSBP17* deficiency triggers precocious fruit ripening, characterized by accelerated chlorophyll degradation, increased soluble sugar accumulation, and significantly reduced fruit firmness.

**Discussion:**

Taken together, the accelerated maturation and associated physiological shifts in MaSBP17 silenced fruit confirm its role as a key negative regulator of the ripening process. Our findings provide a systematic characterization of the banana SBP family and identify *MaSBP17* as a critical genetic target for governing the maturation program. This research establishes a solid foundation for biotechnological efforts to extend fruit shelf life and improve post-harvest quality in the banana industry.

## Introduction

Banana (*Musa* spp.) is a vital global staple providing food and economic security for over 400 million people in the tropics (Wu et al., 2023). As a typical climacteric fruit, banana ripening is governed by a sharp increase in respiration and an autocatalytic burst of ethylene production (Bapat et al., 2010). This physiological transition initiates a complex suite of biochemical modifications, including the enzymatic conversion of starch to soluble sugars, chlorophyll degradation in the peel, and the extensive depolymerization of cell wall polysaccharides (Kuang et al., 2021; Yi et al., 2024). While essential for fruit quality, this rapid post-harvest ripening process leads to precipitous softening and high vulnerability to fungal pathogens such as *Colletotrichum musae*, resulting in post-harvest losses that often exceed 30% of total production (Vieira et al., 2017; Jiang et al., 1999).

Fruit ripening is a complex developmental program coordinated by the synergistic interplay between phytohormonal signaling and massive transcriptional reprogramming (Tang et al., 2020). In climacteric species, this process is triggered by a precise hormonal flux, where abscisic acid (ABA) often acts as a precursor to sensitize tissues, thereby facilitating the autocatalytic burst of ethylene (Kou et al., 2021; Lohani et al., 2004). These hormonal signals are subsequently transduced into large-scale gene expression changes through a hierarchy of transcription factors (TFs), which serve as the primary engines driving the ripening process (Li et al., 2024a). Within this regulatory hierarchy, much attention has focused on well-characterized families such as MADS-box, NAC, and MYB, which typically function as downstream executors, establishing positive feedback loops to accelerate ripening-related metabolism (Li et al., 2022, 2019). Among these, the SQUAMOSA promoter-binding protein-like (SBP/SPL) family occupies an important position, which do not merely respond to hormonal cues but act as pivotal integrators of plant age and reproductive timing (Preston and Hileman, 2013; Wang et al., 2009). The essential role of SBPs in fruit development is best exemplified by the discovery of the *Colorless non-ripening* (CNR) locus in tomato (Manning et al., 2006). An epigenetic mutation in this *SBP* gene results in a total failure of the ripening program, underscoring the indispensable function of SBPs in governing fruit maturation (Lai et al., 2020). However, the function of SBP in the ripening process of *Musa* is still obscure. This deficit significantly impedes our ability to precisely manipulate ripening kinetics through molecular breeding, which is essential for developing banana cultivars with optimized post-harvest performance.

The SBP/SPL transcription factor family features a highly conserved 76-residue SBP domain, which coordinates two zinc ions to facilitate the specific recognition of the GTAC core motif (Song et al., 2016). SBPs function as pivotal regulatory nodes that coordinate critical traits such as flowering time, plant architecture, and organ size, with their functional output being precisely governed by miR156/157-mediated post-transcriptional repression (Song et al., 2016; Wu et al., 2009). Crucially, SBPs also serve as essential regulators of the fruit ripening program. As evidenced by the *CNR* locus in tomato, *SBP-box* genes play a pivotal role in orchestrating the maturation process, whereby their disruption leads to a failure in ripening (Manning et al., 2006). The functional versatility of the SBP family is further demonstrated by its involvement in a diverse array of ripening-related metabolic pathways in other horticultural crops. Beyond tomato, SBPs are critical for traits associated with fruit ripening, such as anthocyanin-mediated coloration and chlorophylls accumulation (Li et al., 2024b, 2020). Given the multiple roles of SBPs in governing fruit maturation across various species, a comprehensive genome-wide identification and characterization of the SBP family in banana (*Musa* spp.) is of critical significance. Elucidating their specific regulatory mechanisms during banana fruit ripening will not only deepen our understanding of the transcriptional networks in climacteric fruits but also provide essential genetic targets for improving fruit quality and post-harvest shelf-life.

In this study, the *SBP* gene family members in banana were systematically identified and analyzed. A total of 57 *MaSBP* genes were identified in the DH-Pahang v4 genome and classified into six distinct groups based on phylogenetic analysis. Integrative analysis of ripening transcriptomes, *cis*-element mapping and qPCR showed that *MaSBP17* expression is significantly downregulated at the onset of fruit ripening. Functional assays using virus-induced gene silencing and physiological profiling revealed that MaSBP17 acts as a negative regulator of the ripening program by modulating soluble sugars, chlorophyll content, reactive oxygen species (ROS) levels, and fruit firmness. These results provide a comprehensive characterization of the banana *SBP* family and establish *MaSBP17* as a potential molecular target for extending fruit shelf-life. Overall, these findings expand the current understanding of the transcriptional regulatory networks governing banana fruit maturation and provide a theoretical framework for deciphering the complex interplay between SBP transcription factors and ripening-related physiological changes.

## Materials and methods

### Identification and physicochemical characterization

The protein sequences of *Musa acuminata* DH-Pahang v4 were retrieved from the Banana Genome Hub (https://banana-genome-hub.southgreen.fr/) (Droc et al., 2022). To identify the SBP family members, the Hidden Markov Model profile for the SBP domain PF03110 was obtained from the Pfam database (http://pfam.xfam.org/) (Mistry et al., 2021) and used as a query for a HMMER v3.3 search with an E-value threshold of 1e^-5^ (Eddy, 2011). All candidate sequences were further validated for domain integrity through the SMART database (https://smart.embl.de/) (Letunic et al., 2021) and NCBI-CDD database (https://www.ncbi.nlm.nih.gov/Structure/cdd/wrpsb.cgi) (Lu et al., 2020) to ensure the presence of the SBP domain. Physicochemical parameters, including theoretical isoelectric point and molecular weight, were predicted using the ProtParam tool from the ExPASy server (https://web.expasy.org/protparam/) (Duvaud et al., 2021).

### Phylogenetic and structural analyses

Multiple sequence alignments of SBP proteins from *Musa acuminata*, *Arabidopsis thaliana*, and *Oryza sativa* were conducted using ClustalW v2.1 with default parameters (Thompson et al., 1994). A phylogenetic tree was constructed using the Maximum Likelihood method in MEGA v11.0.10 (Tamura et al., 2021) with 1,000 bootstrap replicates and the JTT+G model. Conserved protein motifs were identified using the MEME suite v 5.0.4 with the maximum number of motifs set to 10 (Bailey et al., 2009). The exon-intron architectures were determined by comparing the coding sequences with their corresponding genomic sequences and were visualized using the Gene Structure View tool in TBtools v2.76 (Chen et al., 2020).

### *Cis*-acting element and transcriptome analysis

For promoter analysis, the 2,000 bp sequences upstream of the start codon for each *MaSBP* gene were extracted from the banana genome database. The presence of *cis*-acting regulatory elements was predicted using the PlantCARE online server (https://bioinformatics.psb.ugent.be/webtools/plantcare/html/) (Lescot et al., 2002). For expression profiling, transcriptomic data of banana peel and pulp treated with ethephon and 1-MCP were obtained from the TCOD database (https://ngdc.cncb.ac.cn/tcod/) (Kang et al., 2024). To identify significantly differentially expressed genes (DEGs), the expression levels (FPKM) were compared between the treated samples and the Day 0 control. Genes with a ∣log _2_ (FoldChange)∣≥1.0 were defined as significantly regulated members. Expression levels were quantified as Fragments Per Kilobase of transcript per Million (FPKM) mapped reads, and heatmaps were generated using Z-score normalized values via TBtools v2.76 (Chen et al., 2020).

### RT-qPCR analysis

Plant materials of Xiangfen NO.3 banana (AAB) were harvested at 25, 45, 65, and 85 days after male bud removal. Total RNA was extracted from the fruit pulp using the Plant RNA Kit (Tiangen, China). First-strand cDNA was synthesized using the PrimeScript RT Reagent Kit (Takara, Japan). Quantitative real-time PCR was performed using SYBR Green Premix on an ABI 7500 Real-Time PCR System. Specific primers were designed using Primer 5.0 software, and the *MaACTIN* gene was utilized as an internal control for normalization. Relative expression levels were calculated using the 2-^ΔΔCt^ method based on three biological replicates (Livak and Schmittgen, 2001).

### VIGS and physiological measurements

A 300 bp specific fragment of *MaSBP17* was amplified and cloned into the pTRV2 virus vector to generate the VIGS construct according to the previous report (Zhu et al., 2023). *Agrobacterium tumefaciens* strain EHA105 harboring pTRV1 and pTRV2-*MaSBP17* was co-infiltrated into banana fruit fingers at sixty percent maturity. This stage is defined as the late pre-climacteric mature-green phase, occurring approximately 75 days after male bud removal, when the fruit angularity is nearly lost but the peel remains entirely green and the pulp is firm. Fruit firmness was measured in Newtons using a GY-4 digital penetrometer at 5 days post infiltration. Total chlorophyll was extracted with eighty percent acetone and measured spectrophotometrically (Schoefs et al., 2001). Total soluble sugars were determined using the anthrone-sulfuric acid method (Yemm and Williams, 1954). The production rate of reactive oxygen species was quantified using the hydrogen peroxide titanium sulfate method (Patterson et al., 1984).

### Statistical analysis

All experimental data were collected from at least three independent biological replicates and presented as the mean ± SD. Statistical significance was determined using Student’s t-test in GraphPad Prism v9.0 software.

## Results

### Genome-wide identification and characterization of the banana SBP family

**Figure 1:**
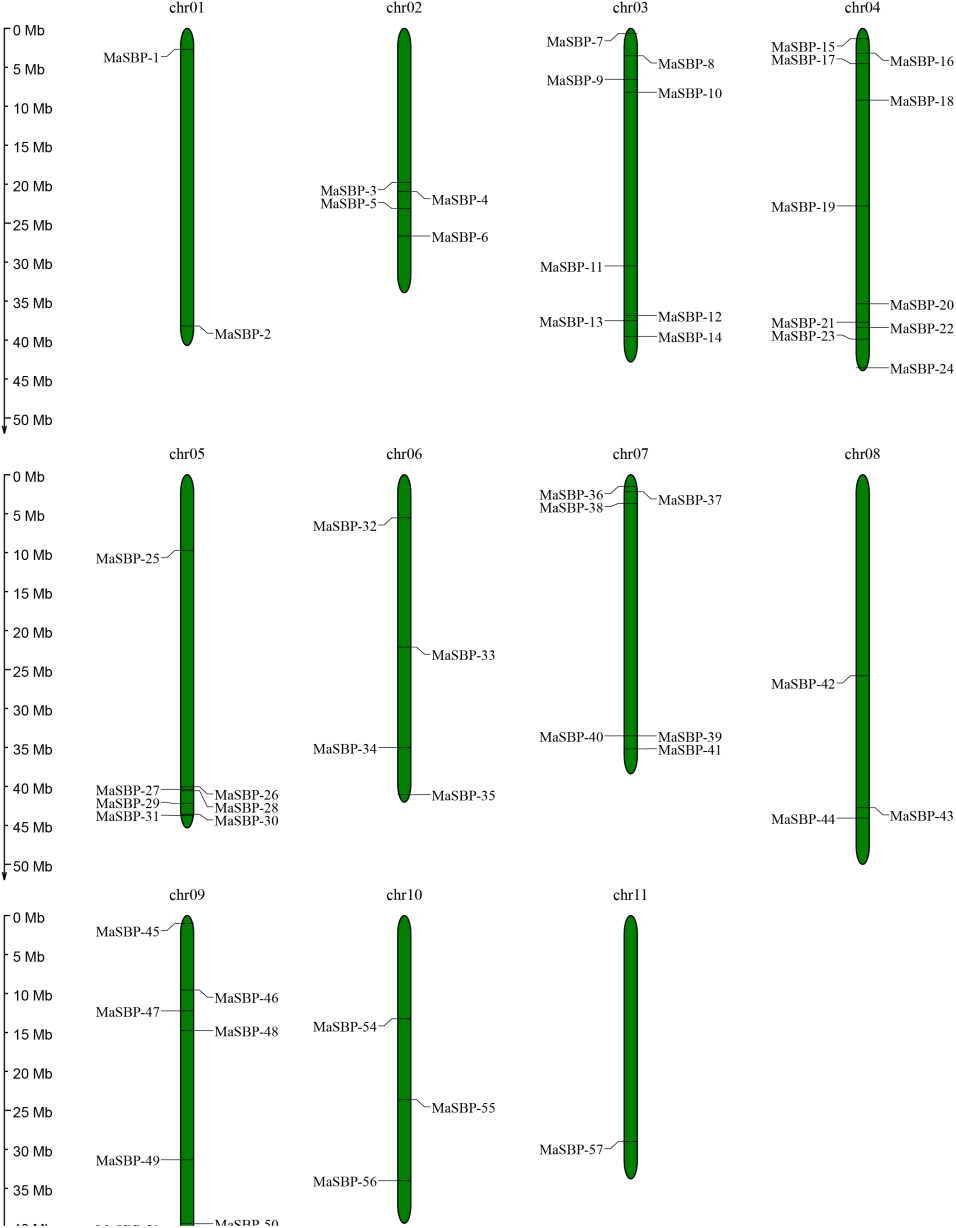
Chromosomal distribution of *MaSBP* genes in banana. The physical positions of 57 *MaSBP* genes are mapped onto the 11 chromosomes (chr01-chr11). Chromosome numbers are indicated at the top of each bar, and the scale on the left represents the chromosomal length in megabases (Mb). Gene names are indicated on both sides of the corresponding chromosomes according to their physical loci.

To identify the *SBP* gene family in banana, a comprehensive genome-wide search of the *Musa acuminata* (DH-Pahang v4) assembly was conducted using HMMER v3.3 and BLASTP v2.12.0+. A total of 57 non-redundant *MaSBP* genes were identified and designated as *MaSBP1* through *MaSBP57* according to their chromosome position (**Table 1**). These genes were non-randomly distributed across all 11 chromosomes, with the highest densities on chromosomes 04 (10 genes) and 09 (9 genes), whereas chromosome 11 contains only a single member (**Figure 1**).

**Table 1:** Genome-wide identification and characterization of the MaSBP gene family in banana.

Name	Gene ID	Genomic location		Chr.	ORF/bp	Exon	AA	pI	MW (kDa)
MaSBP-1	Macma4_01_g04120	2710631	2711693	chr01	564	2	187	9.52	20.82
MaSBP-2	Macma4_01_g26640	39139791	39152894	chr01	1200	3	399	6.93	43.58
MaSBP-3	Macma4_02_g05270	20345949	20357321	chr02	2991	11	996	5.83	110.44
MaSBP-4	Macma4_02_g06500	21555007	21556402	chr02	648	2	215	9.24	23.75
MaSBP-5	Macma4_02_g09590	23774152	23777264	chr02	1182	3	393	6.50	42.56
MaSBP-6	Macma4_02_g15410	27320146	27326594	chr02	1671	12	556	9.38	60.71
MaSBP-7	Macma4_03_g00660	629076	630777	chr03	972	3	323	9.25	35.31
MaSBP-8	Macma4_03_g05540	3639311	3644326	chr03	1395	6	464	9.13	50.69
MaSBP-9	Macma4_03_g09270	6694414	6705301	chr03	2463	10	820	5.65	92.07
MaSBP-10	Macma4_03_g11410	8479309	8485386	chr03	1095	3	364	8.94	38.40
MaSBP-11	Macma4_03_g18590	31240143	31242298	chr03	1053	4	350	5.84	37.98
MaSBP-12	Macma4_03_g26570	37917050	37925244	chr03	2385	10	794	5.95	89.09
MaSBP-13	Macma4_03_g27220	38440885	38450355	chr03	2961	11	986	5.68	109.00
MaSBP-14	Macma4_03_g30390	40618463	40623496	chr03	1410	4	469	8.63	51.39
MaSBP-15	Macma4_04_g01510	1422155	1426098	chr04	1107	3	368	9.26	38.96
MaSBP-16	Macma4_04_g04150	3342931	3344149	chr04	924	3	307	9.16	33.46
MaSBP-17	Macma4_04_g05990	4581898	4586821	chr04	1416	6	471	9.45	51.60
MaSBP-18	Macma4_04_g13010	9489145	9494926	chr04	1272	5	423	9.14	46.69
MaSBP-19	Macma4_04_g18310	23350200	23351766	chr04	1020	3	339	8.39	36.98
MaSBP-20	Macma4_04_g29160	36231423	36233186	chr04	1071	3	356	6.90	39.56
MaSBP-21	Macma4_04_g32230	38649976	38650826	chr04	564	2	187	9.67	20.71
MaSBP-22	Macma4_04_g33280	39317724	39318920	chr04	987	2	328	9.19	35.66
MaSBP-23	Macma4_04_g35760	40928542	40929928	chr04	1044	3	347	9.02	38.18
MaSBP-24	Macma4_04_g42180	44764114	44765956	chr04	516	2	171	9.48	18.98
MaSBP-25	Macma4_05_g13770	9981841	9985666	chr05	1068	3	355	8.84	38.83
MaSBP-26	Macma4_05_g25410	41124138	41135584	chr05	1485	7	494	9.14	54.71
MaSBP-27	Macma4_05_g25680	41413378	41418999	chr05	3276	11	1091	7.88	120.69
MaSBP-28	Macma4_05_g26050	41674593	41679614	chr05	1371	7	456	9.10	49.86
MaSBP-29	Macma4_05_g28440	43271978	43275549	chr05	1176	3	391	6.69	42.34
MaSBP-30	Macma4_05_g30460	44641979	44648664	chr05	2391	11	796	5.79	90.04
MaSBP-31	Macma4_05_g30840	44903440	44911547	chr05	2970	11	989	6.45	109.91
MaSBP-32	Macma4_06_g07880	5592497	5601851	chr06	1110	5	369	7.65	39.95
MaSBP-33	Macma4_06_g23880	22713869	22715953	chr06	1128	3	375	8.03	39.73
MaSBP-34	Macma4_06_g30570	35955998	35957388	chr06	822	3	273	6.79	29.96
MaSBP-35	Macma4_06_g39570	42212601	42222131	chr06	1521	8	506	9.44	56.49
MaSBP-36	Macma4_07_g02010	1570316	1575635	chr07	1134	3	377	9.07	40.22
MaSBP-37	Macma4_07_g02800	2190144	2191397	chr07	720	3	239	6.50	26.74
MaSBP-38	Macma4_07_g05150	3753042	3754527	chr07	1032	3	343	9.61	37.75
MaSBP-39	Macma4_07_g23410	34327242	34330591	chr07	1092	3	363	9.50	38.55
MaSBP-40	Macma4_07_g23520	34422390	34428131	chr07	3297	11	1098	6.68	121.34
MaSBP-41	Macma4_07_g25950	36080600	36085680	chr07	1203	3	400	8.22	43.38
MaSBP-42	Macma4_08_g17020	26566241	26572127	chr08	1206	3	401	6.90	44.05
MaSBP-43	Macma4_08_g25240	43822132	43828875	chr08	1422	7	473	8.83	51.98
MaSBP-44	Macma4_08_g27110	45193678	45200876	chr08	1104	3	367	8.64	39.08
MaSBP-45	Macma4_09_g01380	1079825	1083553	chr09	1179	3	392	8.11	42.68
MaSBP-46	Macma4_09_g14330	9769241	9775951	chr09	1377	9	458	9.14	50.12
MaSBP-47	Macma4_09_g17480	12618942	12632507	chr09	2463	10	820	5.54	91.83
MaSBP-48	Macma4_09_g19220	15082047	15089551	chr09	1071	3	356	9.01	37.80
MaSBP-49	Macma4_09_g21270	32125223	32127429	chr09	1119	3	372	8.56	41.04
MaSBP-50	Macma4_09_g24190	40606548	40608342	chr09	1032	3	343	8.68	36.83
MaSBP-51	Macma4_09_g25000	41317217	41322303	chr09	1323	6	440	8.06	48.56
MaSBP-52	Macma4_09_g29910	45359810	45363469	chr09	1110	3	369	8.48	39.36
MaSBP-53	Macma4_09_g31600	46516925	46518966	chr09	1116	2	371	10.20	41.45
MaSBP-54	Macma4_10_g06830	13521635	13530534	chr10	1422	6	473	9.15	52.07
MaSBP-55	Macma4_10_g11050	24333643	24334715	chr10	687	2	228	9.47	25.63
MaSBP-56	Macma4_10_g27200	34951297	34957881	chr10	1332	6	443	9.08	48.96
MaSBP-57	Macma4_11_g18990	29821305	29824782	chr11	1041	4	346	9.39	38.39

Structural analysis revealed substantial diversity in the *MaSBP* gene architecture (**Table 1**). The open reading frames (ORFs) range from 516 to 3,297 bp, encoding proteins of 171 to 1,097 amino acids (AA) with an average length of 459 AA. The exon-intron organization varied significantly, with exon numbers ranging from 2 to 12. While 43.86% of the family members contained three exons, a subset of ten genes (including *MaSBP3, 6, 9, 12, 13, 27, 30, 31, 40*, and *47*) exhibited complex structures with 10–12 exons, suggesting potential functional diversification through alternative splicing.

Predicted physicochemical properties indicated that the molecular weights (MW) of MaSBP proteins ranged from 18.98 to 121.34 kDa, with theoretical isoelectric points (pI) ranging from 5.54 to 10.20. The majority of the members (41/57) are basic (pI > 7.0), while 16 members are acidic (pI < 7.0). This clustering of high molecular weight, high structural complexity, and high pI suggested that these members may form a clear functional divergence of these large-molecular-weight members.

In summary, the structural and physicochemical heterogeneity of the MaSBP family provides a robust genomic resource for further functional dissection of the SBP gene family in banana.

### Chromosomal distribution of the *MaSBP* gene family

To delineate the spatial organization of the *MaSBP* family, the physical positions of the 57 members were mapped across the 11 chromosomes of the *Musa acuminata* genome. The *MaSBP* genes exhibited a non-random distribution, with significant variation in gene density among chromosomes (**Figure 1**). A higher abundance of *MaSBP* genes were observed on chromosomes 03, 04, and 09, which collectively contained 27 members, accounting for 47.37% of the entire family. In contrast, chromosomes 01, 08, 10, and 11 displayed relatively few *MaSBP* members, containing only 2, 3, 3, and 1 members, respectively. This heterogeneous distribution suggested that the expansion of the *MaSBP* family may be influenced by regional differences in chromosomal recombination frequencies. Notably, several members, including *MaSBP2, MaSBP24*, and *MaSBP53*, were localized to distal telomeric or sub-telomeric regions. These regions are typically characterized by elevated recombination rates and transposable element activity, which may potentially facilitate gene family diversification through non-allelic homologous recombination. Furthermore, the inherent instability of telomeric regions suggested that these specific members may be subject to more complex epigenetic regulation. Collectively, the uneven chromosomal distribution and localized clustering provide crucial insights into the evolutionary expansion and potential functional specialization of the *MaSBP* family in banana development.

### Phylogenetic analysis of the *MaSBP* gene family

**Figure 2:**
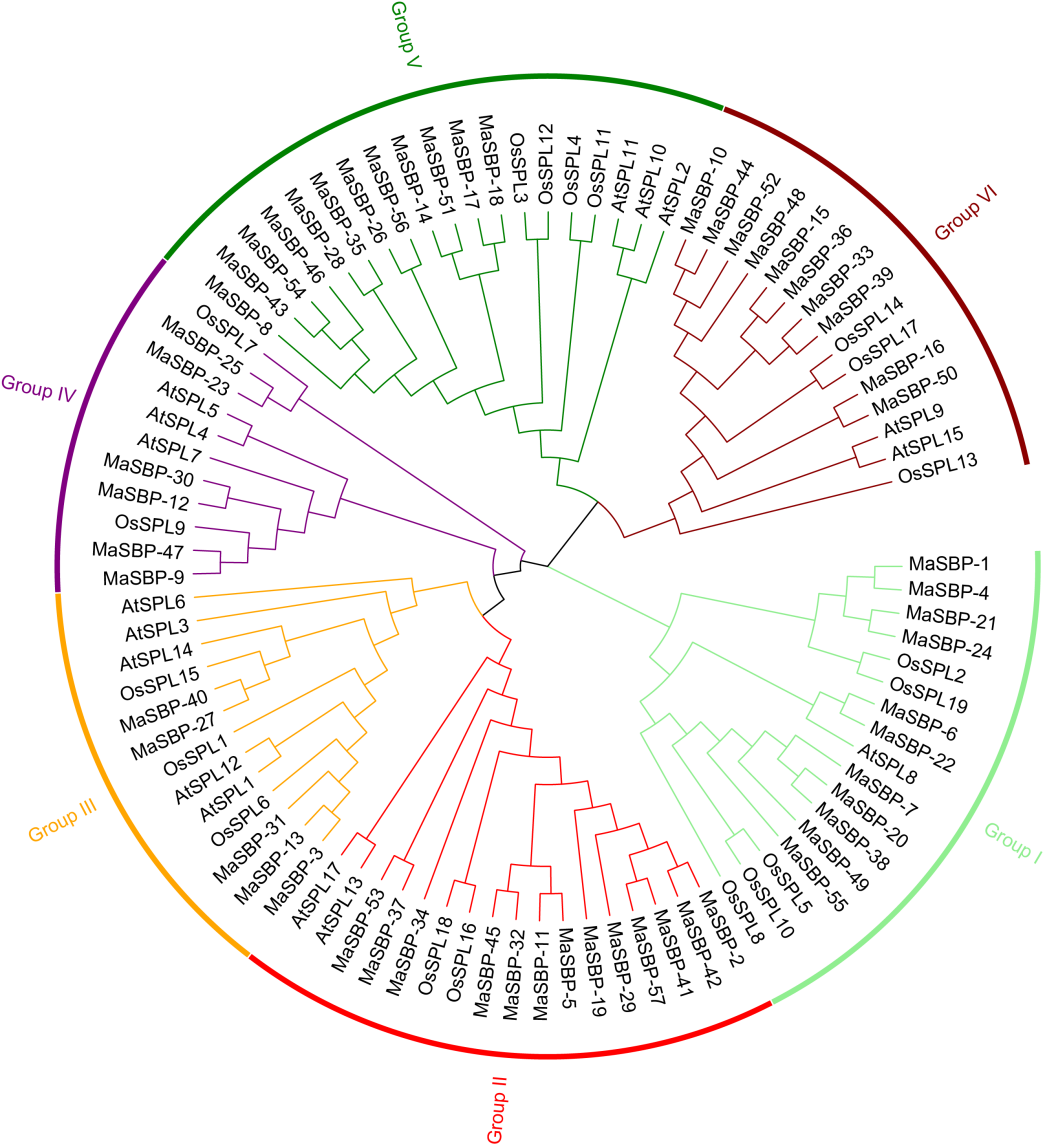
Phylogenetic relationship of SBP proteins from banana, *Arabidopsis*, and rice. The Maximum Likelihood (ML) tree was constructed using protein sequences of 57 MaSBPs from *Musa acuminata* (banana), 16 AtSPLs from *Arabidopsis thaliana*, and 19 OsSPLs from *Oryza sativa*. The tree was generated using MEGA v11.0.10 with 1,000 bootstrap replicates. The SBP family is categorized into six major subgroups (Group I-VI), distinguished by different colored branches and outer categorical labels. The prefixes ‘Ma’, ‘At’, and ‘Os’ denote *Musa acuminata*, *Arabidopsis thaliana*, and *Oryza sativa*, respectively.

To elucidate the evolutionary relationships and functional divergence of the banana *MaSBP* family, a Maximum Likelihood (ML) phylogenetic tree was constructed using SBP protein sequences from *Musa acuminata* (Ma), *Arabidopsis thaliana* (At), and *Oryza sativa* (Os). The phylogenetic analysis subdivided the SBP members into six distinct clades, designated as Groups I to VI (**Figure 2**). Group I contained 11 MaSBP members (MaSBP1, 4, 6, 7, 20, 21, 22, 24, 38, 49, and 55), which clustered closely with AtSPL8 and OsSPL2/5/8/10/19. This high degree of conservation indicated that these genes likely derived from an ancestral copy present before the divergence of monocots and dicots, potentially maintaining core roles in plant growth and development. Similarly, Groups II, III, IV, V, and VI harbored 13, 5, 6, 12, and 10 banana SBP members, respectively. Notably, Group II exhibits a significant lineage-specific expansion in banana, which far exceeds the number of homologs in rice and *Arabidopsis*. Furthermore, in Group V, the banana *MaSBP* genes formed a distinct subclade separate from their *Arabidopsis* and rice counterparts. This divergence suggested that these members may have evolved specialized functions in banana that are distinct from those in other species. Collectively, the presence of SBPs from all three species in each clade, coupled with the expansion of MaSBP members, indicates a conserved origin followed by lineage-specific diversification.

### Conserved motif and gene structure analysis of the *MaSBP* family

**Figure 3:**
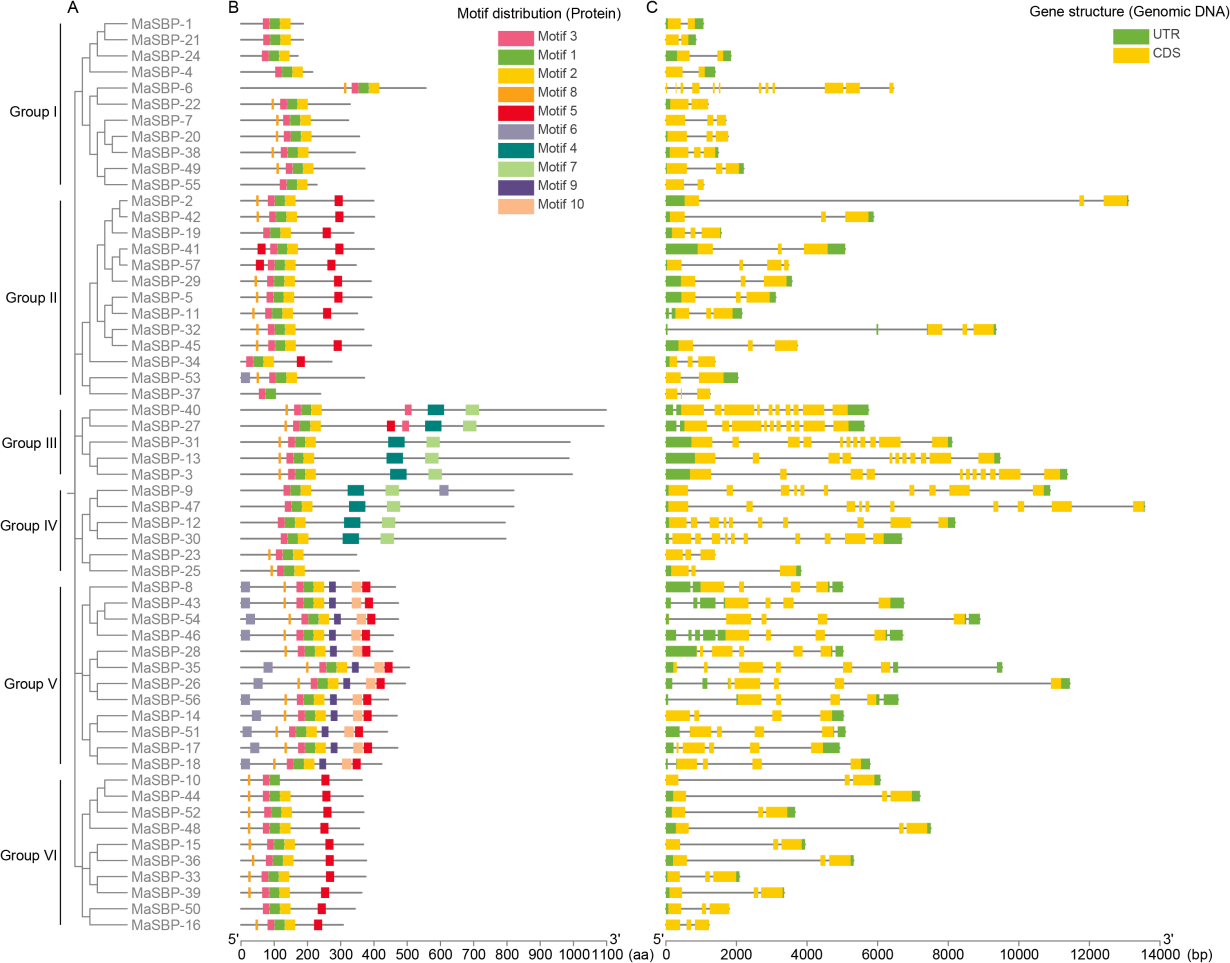
Conserved motifs and gene structures of the *MaSBP* family. **(A)** Phylogenetic tree of 57 *MaSBP* proteins. **(B)** Distribution of 10 MEME-identified motifs. Each motif is depicted by a distinct colored box, with relative positions scaled to protein length. **(C)** Gene structural features of *MaSBP* genes. Coding sequences (CDS), untranslated regions (UTRs), and introns are represented by yellow boxes, green boxes, and black lines, respectively. The scales at the bottom indicate protein length (amino acids) and genomic DNA length (base pairs).

To explore the structural diversity and functional specialization of the *MaSBP* family, conserved protein motifs were identified using the MEME suite v5.0.4 and integrated with their respective exon-intron architectures. A total of 10 distinct conserved motifs (Motifs 1-10) were identified (**Figure 3**). Motifs 1 and 3 were present in all 57 *MaSBP* members, suggesting they represent the essential components of the SBP DNA-binding domain. Beyond these core motifs, the distribution of other motifs were highly clade-specific. For example, Group I members exhibited a highly uniform arrangement consisting primarily of Motifs 1, 2, and 3. In contrast, Groups II and VI were characterized by the presence of Motifs 5 and 8, while Group V possessed the most complex motif composition (including Motifs 6, 9, and 10), reflecting significant functional divergence among these subfamilies.

The exon-intron organization further supports the phylogenetic classification of the *MaSBP* family. Members of Groups I, II, and VI were structurally simple, typically harboring 2–3 exons. Conversely, Groups III and IV exhibited much higher structural complexity, with most members containing more than 10 exons. Such architectural diversity suggested that these specific groups may achieve functional versatility through mechanisms such as alternative splicing or differential mRNA stability. Group V members displayed a moderate structure, consistently containing 4–6 exons.

### Analysis of *cis*-acting elements in the *MaSBPs* promoter regions

**Figure 4:**
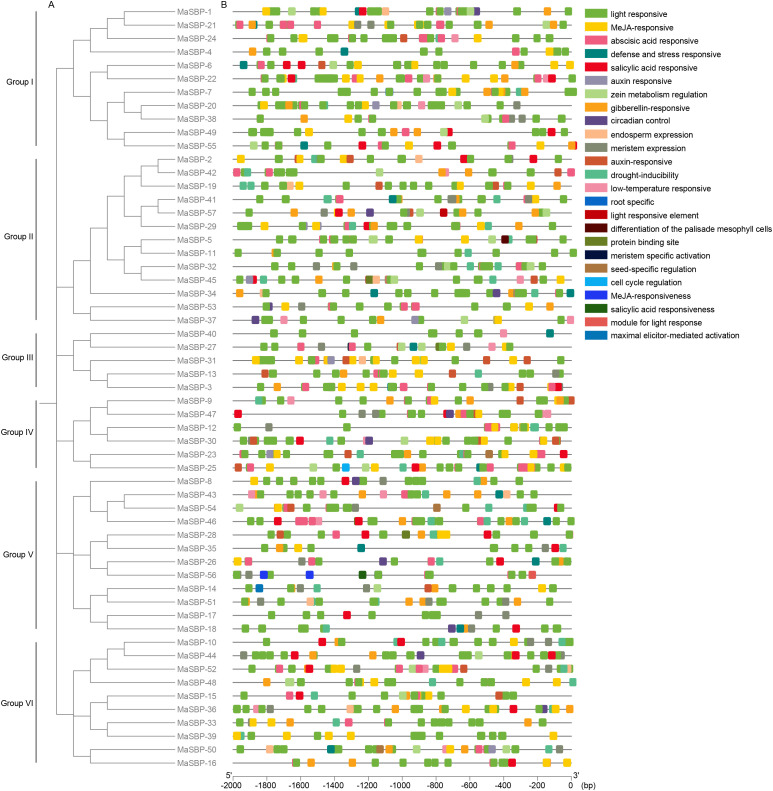
Predicted *cis*-acting elements in the promoter regions of banana *MaSBP* genes. **(A)** Phylogenetic tree of the 57 *MaSBP* members, providing the evolutionary context for the promoter analysis. **(B)** Schematic distribution of 25 functional *cis*-acting elements identified within the 2,000 bp upstream regions from the start codon (ATG). Each element is represented by a specific colored symbol as defined in the legend on the right. The scale at the bottom indicates the promoter location in base pairs (bp).

To investigate the potential regulatory mechanisms of *MaSBPs* in plant development and environmental adaptation, *cis*-acting elements were predicted within the 2.0 kb upstream promoter regions of the 57 genes using the PlantCARE database. A total of 25 functional categories were identified across the family (**Figure 4**). Among the 1,509 total elements detected, light-responsive (46%), MeJA-responsive (15%), and abscisic acid (ABA)-responsive (12%) elements were the most abundant. The distribution of these major elements exhibited high consistency across most phylogenetic subfamilies. Among the total detected elements, light-, MeJA-, and ABA-responsive elements were identified as the top three most frequent motifs in Groups I, II, III, IV, and VI (**Supplementary Table 2**). For instance, in Group I, these elements accounted for 59.77%, 23.44%, and 16.80% of the subfamily-specific repertoire, respectively. In contrast, Group V exhibited a distinct regulatory signature, where gibberellin-responsive elements (7.03%) were more prevalent than MeJA-responsive elements (5.47%), ranking among the top three categories alongside light- and ABA-responsive motifs (**Supplementary Table 2**). The enrichment of these motifs suggests that the *MaSBP* family functions as a central hub for integrating light signaling, circadian rhythms, and multiple phytohormone pathways to coordinate banana maturation and stress responses, particularly MeJA and ABA.

### Expression profiles of *MaSBP* genes in response to ethylene and 1-MCP treatments

**Figure 5:**
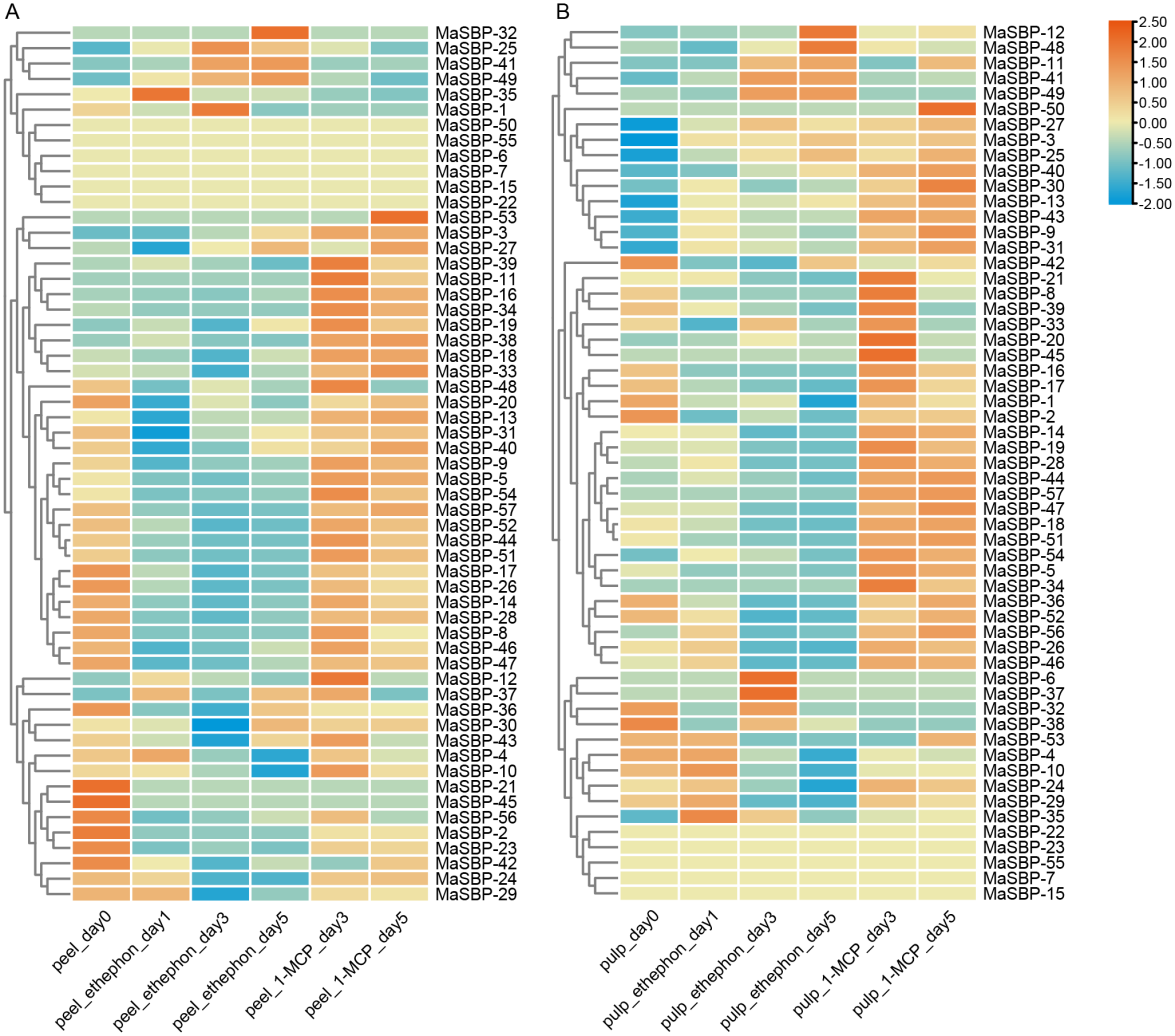
Expression profiles of *MaSBP* genes in banana peel and pulp under ethylene and 1-MCP treatments. The heatmaps illustrate the relative expression levels (Z-score normalized) of 57 *MaSBPs* genes in the **(A)** peel and **(B)** pulp at different stages. Day 0 (control), Ethylene treatment (Days 1, 3, and 5), and 1-MCP treatment (Days 3 and 5). The color scale on the top right represents the expression level, ranging from blue (low expression) to orange/red (high expression). Hierarchical clustering was performed to group genes with similar expression patterns.

To elucidate the regulatory roles of *MaSBPs* in banana fruit maturation, their expression patterns were analyzed using transcriptomic data from the TCOD database, covering peel and pulp tissues treated with ethphon (an ethylene releaser) and 1-MCP (an ethylene receptor antagonist). The heatmaps revealed divergent transcriptional responses among family members, suggesting functional specialization during the ripening process (**Figure 5**). A large group of 26 *MaSBP* members (including *MaSBP3, 5, 8, 9, 13, 14, 16, 17, 18, 19, 20, 26, 27, 28, 31, 33, 34, 39, 40, 44, 46, 47, 51, 52, 54*, and *57*) exhibited significant up-regulation following 1-MCP treatment in both peel and pulp (log 2 FC≥1.0, **Supplementary Table 3**). Notably, in the pulp, *MaSBP5* and *MaSBP54* showed 6.31-fold and 5.22-fold increases, respectively, at 3 days post 1-MCP treatment compared to the pre-treatment stage (Day 0). Since 1-MCP effectively delays ripening by blocking ethylene signaling, the high expression of these genes identifies them as potential negative regulators (repressors) of the banana ripening program. *MaSBP32* and *MaSBP35* exhibited a different expression trend, with a transient or minor induction following ethylene treatment in certain tissues. However, due to their relatively low absolute transcript abundance (FPKM < 1), their potential function in fruits development requires further experimental validation. The remaining 29 *MaSBPs* members displayed either constitutive expression with no significant fluctuations across treatments or maintained extremely low transcript levels in both fruit tissues. This suggests that these genes may not be directly involved in the ethylene-mediated ripening program, but rather participate in other biological processes, such as vegetative growth, floral organ development, or responses to other environmental stimuli (Wu et al., 2009; Wang et al., 2009; Preston and Hileman, 2013; Song et al., 2021). Collectively, these contrasting expression patterns provide a robust foundation for selecting key candidates, particularly the repressor-like *MaSBPs*, to manipulate banana ripening and extend post-harvest shelf-life.

### Expression patterns of representative *MaSBPs* genes during fruit development

**Figure 6:**
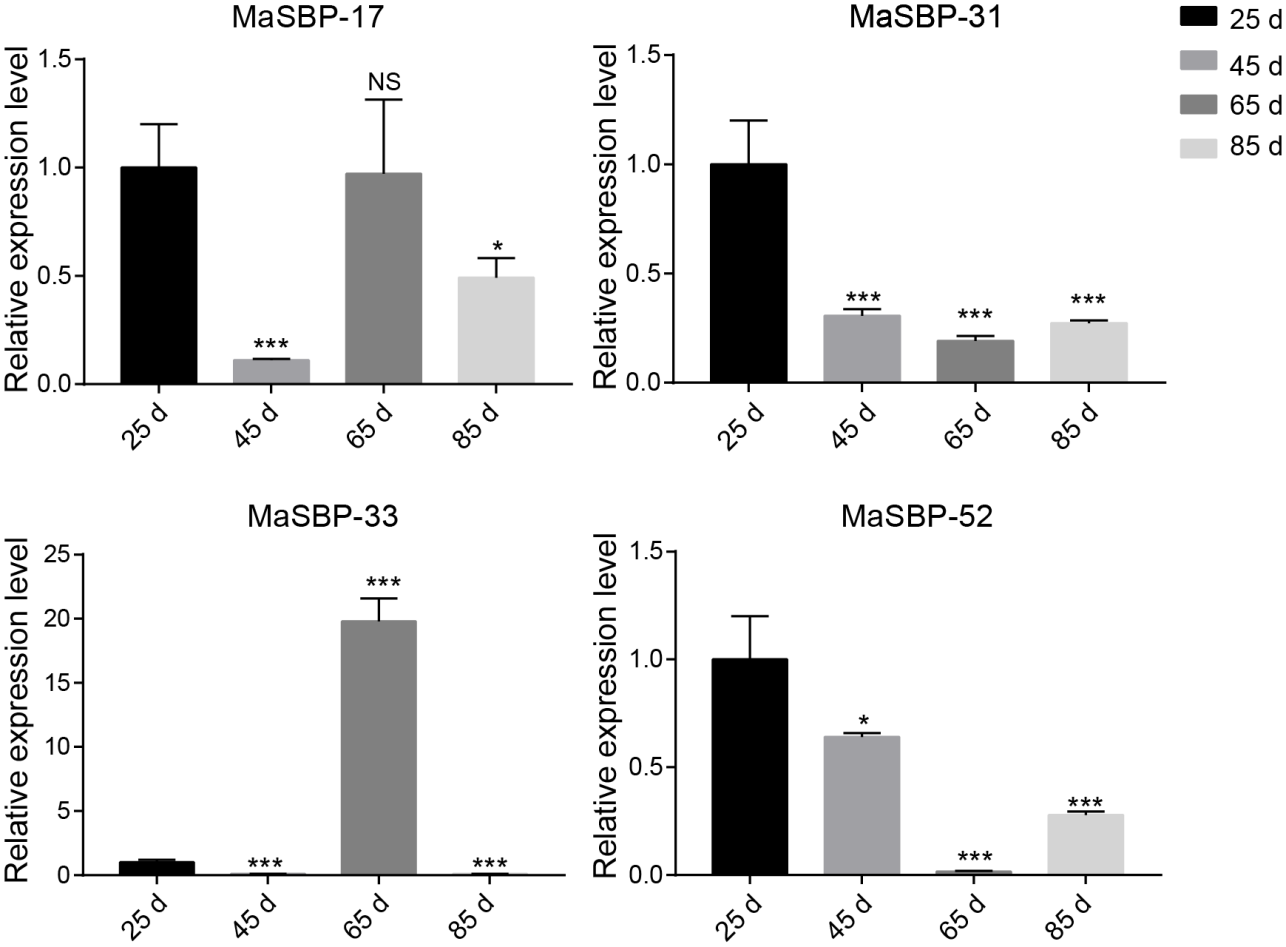
Expression patterns of four representative *MaSBPs* genes during banana fruit development. Transcript levels of *MaSBP17, 31, 33*, and *52* were determined by RT-qPCR in fruit harvested at 25, 45, 65, and 85 days after flowering (DAF). These sampling points represent the young fruit stage, mid-developmental starch accumulation stage, pre-mature stage, and mature-green stage, respectively. Specifically, 25 DAF is the period of rapid growth, while 45 DAF is characterized by intense nutrient storage. The 65 DAF point marks the conclusion of fruit filling, and 85 DAF represents the stage of physiological maturity at the threshold of the ripening transition. The relative expression levels were calculated using the 2^−ΔΔCt^ method, with the 25 DAF stage serving as the control (set to 1.0). Data represent the mean ± SD of three biological replicates. Asterisks indicate significant differences compared to the 25 DAF stage according to Student’s t-test (*P < 0.05; ***P < 0.001; NS, non-significant).

To identify potential negative regulators of banana ripening, we selected candidate genes showing higher expression under 1-MCP than ethylene treatment, focusing on Groups III, V, and VI subfamilies with the most conserved motifs. This integrated screening identified *MaSBP17, 31, 33*, and *52* as key targets for RT-qPCR validation. To further validate their functions in coordinating banana maturation, RT-qPCR was performed to analyze the expression profiles of the four representative members (*MaSBP17, 31, 33*, and *52*) at four critical developmental stages (25, 45, 65, and 85 days after flowering (DAF)). The results demonstrated that these genes exhibit distinct and dynamic transcriptional signatures as the fruit progresses toward maturity (**Figure 6**). Using the 25 DAF stage as a reference, *MaSBP31* and *MaSBP52* transcript levels dropped sharply after the early stage and remained at basal levels throughout late development. In contrast, *MaSBP17* and *MaSBP33* exhibited a characteristic fluctuating pattern. Specifically, *MaSBP17* transcript levels dropped sharply at 45 DAF, recovered to near-basal levels (comparable to 25 DAF) at 65 DAF, and then significantly declined again at the onset of ripening (85 DAF). *MaSBP33* followed a similar trend but showed a much more intense induction at 65 DAF (~20-fold). While *MaSBP31* and *MaSBP52* remained down-regulated after 45 DAF, *MaSBP17* and *MaSBP33* were highly active during the late pre-climacteric stage at 65 DAF. This sustained suppression of *MaSBP31* and *MaSBP52* across all developmental stages suggests they function in early fruit growth rather than the final ripening transition, while the extreme fluctuation of *MaSBP33* likely reflects a specialized metabolic response. Conversely, *MaSBP17* uniquely recovered at 65 DAF before dropping sharply at the 85 DAF ripening threshold, identifying it as a potential repressor against fruits maturation. Consequently, *MaSBP17* was prioritized as the primary candidate for fruits maturation in banana.

### MaSBP17 negatively regulates banana fruit maturation by modulating fruit softening and ROS homeostasis

**Figure 7:**
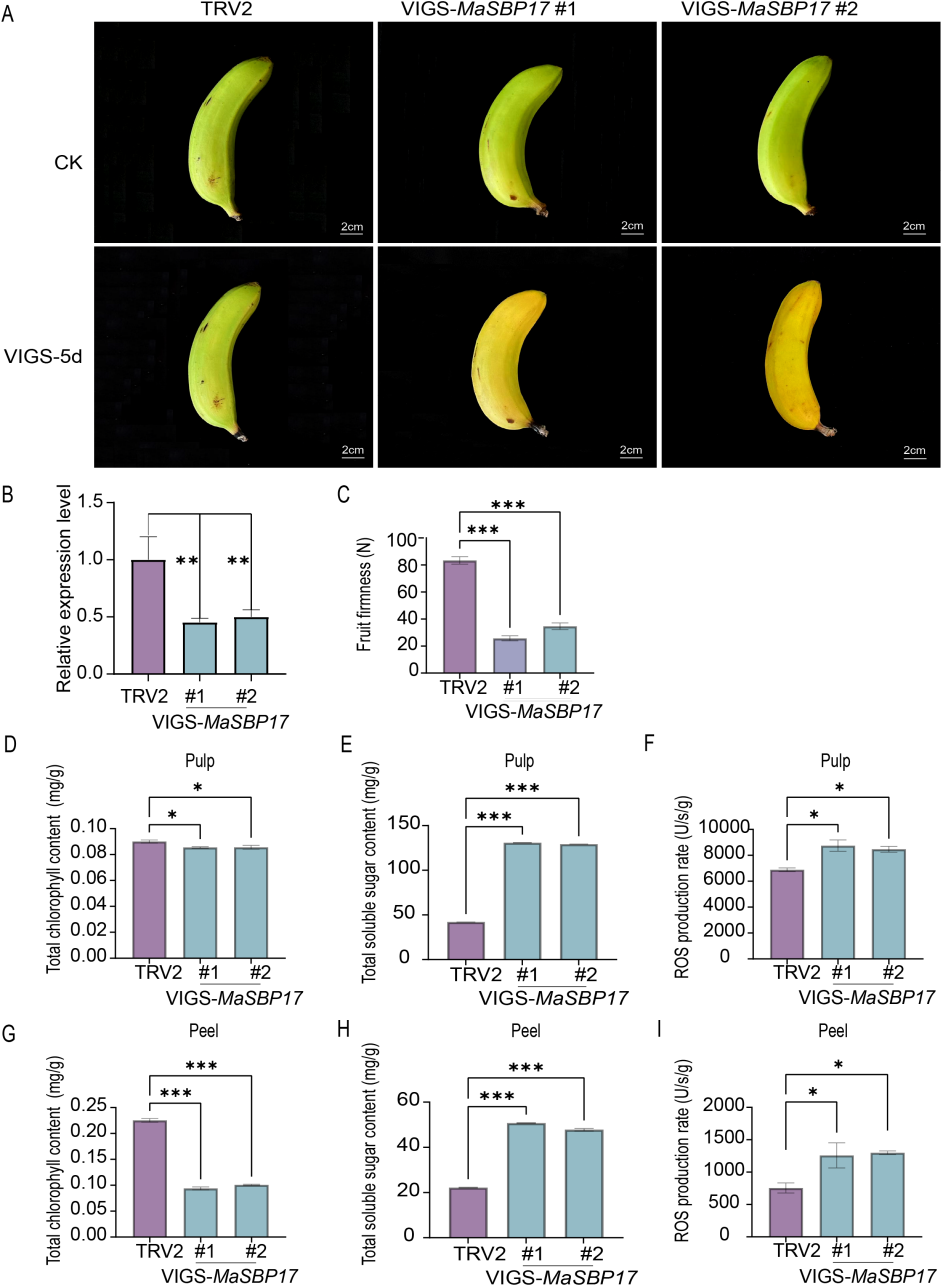
Functional characterization and physiological profiling of *MaSBP17* in banana fruit via virus induced gene silencing. **(A)** The visual phenotype of banana fruit at five days after infiltration with TRV2 and two independent VIGS-*MaSBP17* constructs. **(B)** The silencing efficiency of *MaSBP17* determined by RT-qPCR. **(C)** The fruit firmness measured in Newtons. **(D, G)** The total chlorophyll content in the pulp and peel respectively. **(E, H)** The total soluble sugar content in the pulp and peel. **(F, I)** The ROS production rate in the pulp and peel respectively. Data are presented as the mean ± SD of three biological replicates. Asterisks indicate significant differences between the silenced fruit and the TRV2 control according to Student’s t-test (*represents *P* < 0.05, **represent *P* < 0.01, ***represent *P* < 0.001). The 2 cm scale bars provide a physical reference for the fruit size.

To confirm the specific functional role of *MaSBP17* in coordinating fruit maturation, we performed virus induced gene silencing (VIGS) in banana fruit at the sixty percent maturity stage. This specific developmental point corresponds to the mature-green stage at approximately 75 days after male bud removal, which provides a highly responsive physiological period for observing the transition to ripening. The silencing efficiency was first confirmed by RT-qPCR which showed that the transcript levels of *MaSBP17* in the silenced lines were significantly reduced by approximately fifty percent compared to the TRV2 empty vector control (**Figure 7B**). At five days post infiltration, the *MaSBP17* silenced fruit exhibited a clear premature ripening phenotype characterized by accelerated yellowing of the peel when compared to the control fruit (**Figure 7A**). This visual maturation was accompanied by a dramatic reduction in fruit firmness (**Figure 7C**), indicating that the degradation of cell wall components may be substantially more advanced in the silenced fruit. Comprehensive physiological profiling of both pulp and peel tissues further supported these observations. Consistent with the early yellowing of the fruit, the total chlorophyll content in both tissues was significantly lower in the *MaSBP17* silenced lines than in the TRV2 control (**Figures 7D, G**). Conversely, the levels of total soluble sugars were markedly higher in the silenced fruit, suggesting that the suppression of *MaSBP17* released the inhibition of sugar accumulation (**Figures 7E, H**). Furthermore, the rate of reactive oxygen species (ROS) production was significantly elevated in the silenced fruit (**Figures 7F, I**). Collectively, these results demonstrate that *MaSBP17* functions as a potent negative regulator of banana maturation by inhibiting fruit softening, soluble sugar accumulation, and ROS mediated signaling.

## Discussion

### Evolutionary drivers of the expanded *MaSBPs* repertoire in banana

Banana fruit ripening is a complex and highly coordinated physiological program that critically determines fruit quality, flavor, and post-harvest shelf life (Song et al., 2023; Yang et al., 2022). Previous research in multiple horticultural crops has shown that SBP transcription factors serve as essential master regulators in governing these developmental transitions and metabolic shifts (Manning et al., 2006; Li et al., 2024b, 2020). To gain a more systematic understanding of the regulatory roles that SBPs play specifically during banana maturation, we performed a comprehensive genome-wide analysis of this family. The identification of 57 *MaSBPs* members in the *Musa acuminata* DH-Pahang v4 genome represents a significant update to the previous reports of 41 genes identified in earlier assemblies (Mouri and Islam, 2025). The additional *MaSBP* members identified in the v4 assembly establish a complete and well-defined genomic framework for functional analysis. This ensures that the entire regulatory network governing banana maturation is systematically investigated without overlooking any critical *SBP* members. Besides, the non-random distribution of MaSBPs, particularly the high-density clusters on chromosomes 04 and 09 (**Figure 1**), is likely a result of the whole-genome duplication events that have shaped the *Musa* lineage (D'Hont et al., 2012). Our phylogenetic analysis supports this by showing that Groups II and V contain a disproportionately high number of banana-specific paralogs compared to the corresponding subfamilies in *Arabidopsis* and rice (**Figure 2**). These results indicate that ancient duplications primarily facilitated the expansion of Groups II and V through selective retention. Consequently, these specific lineages developed a significantly larger number of members compared to other subfamilies. Such genomic plasticity allows for the functional diversification of the *MaSBP* family, enabling different members to specialize in complex processes such as fruit maturation and stress adaptation.

### Structural diversity and cross-talk in transcriptional regulation

The significant variation in exon-intron organization among subfamilies provides a structural basis for their functional divergence (**Figure 3**). Specifically, the simple gene architectures found in the majority of subfamilies facilitate rapid transcription and immediate protein synthesis, which is often required for maintaining basal growth and development. This high efficiency occurs because fewer introns reduce the energetic cost and time required for pre-mRNA processing (Castillo-Davis et al., 2002; Choquet et al., 2025). In contrast, the complex exon-intron organization in Groups III and IV suggests a shift toward more intricate regulatory mechanisms. Numerous introns provide the necessary structural framework for alternative splicing which can produce multiple mRNA isoforms and proteins with distinct activities or cellular localizations (Nilsen and Graveley, 2010; Staiger and Brown, 2013). This structural flexibility enables these specific subfamilies to achieve more comprehensive spatial and temporal control, which is essential for managing intricate physiological transitions such as fruit ripening or responses to environmental signals. Therefore, the structural diversification of the *MaSBP* family likely reflects an evolutionary strategy to expand its regulatory repertoire and accommodate the complex developmental programs in banana.

### MaSBP17 functions as a transcriptional repressor to coordinate banana ripening through ROS and fruit softening

The maturation of climacteric fruits is a strictly controlled process that depends on the de-repression of specific transcriptional pathways. This regulatory model is exemplified by the tomato *CNR* locus where SBP-box gene activity serves as a primary trigger for the ripening program (Manning et al., 2006). Our findings align with this mechanism as the transcriptome analysis identified a prominent group of 26 *MaSBP* genes that were significantly induced by the ethylene antagonist 1-MCP (**Figure 5**). Among these candidates, *MaSBP17* was identified as a key regulator due to its unique expression trajectory where it recovered to high levels at 65 DAF but dropped sharply at the 85 DAF ripening threshold (**Figure 6**). This precise downregulation at the onset of maturation suggests that *MaSBP17* must be suppressed to release the inhibition on the ripening cascade. Functional validation through VIGS-mediated silencing further confirmed this hypothesis, as the knockdown of *MaSBP17* led to a significant premature ripening phenotype (**Figure 7**). This proves that the presence of *MaSBP17* is essential to delay maturation until the appropriate developmental stage. Beyond timing the ripening onset, our physiological data demonstrate that MaSBP17 coordinates multiple essential aspects of the maturation program including fruit firmness and oxidative status. The dramatic reduction in fruit firmness observed in the silenced lines indicates that this transcription factor may inhibit the expression of cell wall modifying enzymes to maintain structural integrity, which is consistent with the previous research (Ali et al., 2022). This mechanism is further supported by the ROS surge observed in the *MaSBP17* silenced fruit (**Figures 7F, I**) which correlates with the significant reduction in fruit firmness (**Figure 7C**). This relationship aligns with established models where elevated ROS levels function as secondary messengers to promote cell wall loosening and senescence (Wang et al., 2025). Together, these findings suggest that MaSBP17 regulates fruit softening by simultaneously suppressing enzymatic cell wall degradation and oxidative signaling pathways. This research provides a practical foundation for improving fruit quality and extending shelf life in the global banana industry.

## Data Availability

The datasets presented in this study can be found in online repositories. The names of the repository/repositories and accession number(s) can be found in the article/**Supplementary Material**.
